# Combination of SGLT2 Inhibitors and Loop Diuretics in the Treatment of Heart Failure

**DOI:** 10.3390/jpm15030099

**Published:** 2025-03-03

**Authors:** Yoshiki Murakami, Shunsuke Kiuchi, Shinji Hisatake, Takanori Ikeda

**Affiliations:** Division of Cardiovascular Medicine, Department of Internal Medicine, Toho University Graduate School of Medicine, Tokyo 1438541, Japan; yoshiki.murakami@med.toho-u.ac.jp (Y.M.);

**Keywords:** dapagliflozin, loop diuretics, heart failure

## Abstract

**Background:** Administration of SGLT2 inhibitors leads to a reduction in the dosage of loop diuretics in heart failure (HF) patients; however, it is unclear in what patients the dosage can be reduced. We investigated the factors related to the reduction in loop diuretics in patients who have started receiving dapagliflozin, an SGLT2 inhibitor. **Methods:** In total, 126 consecutive patients with HF who received dapagliflozin for HF at our institution between December 2020 and March 2022 were enrolled. We investigated the change in the dosage of diuretics at the time of dapagliflozin administration and after 6 months and evaluated factors at the time of dapagliflozin initiation that were associated with the dosage of loop diuretic reduction. **Results:** The median of loop diuretics dosage (oral furosemide equivalent) at the time of dapagliflozin administration was 20 mg/day (the mean dosage; 29.5 ± 26.5 mg/day), and after 6 months it decreased to 10 mg/day (the mean dosage; 14.5 ± 15.9 mg/day) (*p* < 0.001). Multivariate analysis showed that the three factors of in-hospital start of dapagliflozin, % patients on β-blockers, and the dosage of loop diuretics independently predicted the reduction in loop diuretic dosage. Even in analyses excluding patients who initiated dapagliflozin during hospitalization, loop diuretic dosage independently predicted loop diuretic reduction in multivariate analysis. The receiver operating characteristic curve for predicting reduced loop diuretic showed that the cut-off value for loop diuretic at the time of administration of dapagliflozin was 20 mg/day of oral furosemide equivalent. **Conclusions:** The dosage of loop diuretic used when dapagliflozin was started is a factor that predicts a subsequent reduction in the dose of loop diuretics.

## 1. Introduction

The number of heart failure (HF) patients is on the rise worldwide, and there are 6.7 million HF patients in the United States [1]. The number of HF patients in Japan is also expected to reach 1.3 million in 2030 [2], and this is worldwide problem that has been called a HF pandemic. Therefore, numerous medications are being used clinically to improve the prognosis of HF; however, the majority of them are only applicable to HF reduced ejection fraction (HFrEF). On the other hand, in Japan, which is an facing aging society, the number of patients with HF who have preserved EF (HFpEF) is on the rise [3]. For HFpEF, the guidelines do not list any medications that improve the prognosis of HF. However, following the results of the DELIVER trial [4] and the EMPEROR-Preserved trial [5], the use of sodium/glucose cotransporter (SGLT) 2 inhibitors (dapagliflozin or empagliflozin) for HFpEF was approved in Japan. SGLT2 inhibitors have been shown to be effective in all EF types of HF [6], including the results of the DAPA-HF trial [7] and the EMPEROR-Reduced trial [8], which were conducted for HFrEF. The mechanisms underlying these benefits of SGLT2 inhibitors for HF are not well understood; however, diuretic properties may contribute to this. The addition of an SGLT2 inhibitor to a loop diuretic causes significant natriuresis [9]. In hospitalized patients, the early introduction of dapagliflozin after admission has been reported to increase urinary volume and shorten hospital stay [10]. The addition of empagliflozin to outpatients of HF reduced the diuretic dosage in 23.1% of patients and reduced the mean loop diuretic dosage by approximately half [11]. However, it is unclear in what patients loop diuretics can be reduced. We investigated changes in diuretic doses after dapagliflozin introduction in patients with HF.

## 2. Materials and Methods

The present study was a single-center retrospective observational study in accordance with the Declaration of Helsinki (patients’ medical records were accessed for data collection) and was approved by the Ethics Committee of Toho University Omori Medical Center (approval number: M23035, approval date: 2 June 2023). The details of the present study were disclosed in an opt-out format on the website of our institution and our department (Department of Cardiovascular Medicine) (granted a waiver for informed consent from study participants). The subjects of the present study were given the opportunity to decline to be enrolled.

### 2.1. Study Participants

In total, 126 consecutive patients (aged 32–94 years) with HF who received dapagliflozin for HF at our institution between December 2020 and March 2022 were enrolled in the present study. HF was diagnosed based on the Framingham criteria or the guidelines of the American Heart Association or the European Society of Cardiology [12,13,14]. In Japan, dapagliflozin is indicated for diabetes (approved in May 2014), HF (approved in November 2020), and chronic kidney disease (approved in August 2021); however, the dosage vary depending on the indication. In the present study, the dosage of dapagliflozin administered to patients was 10 mg daily, which is indicated for HF. In addition, it was confirmed that the enrolled patients had been administered dapagliflozin for HF. Patients with pacemakers were excluded because their vital signs could not be accurately assessed. In order to evaluate the dosage of concomitant diuretics after 6 months, patients in which evaluation 6 months after administration of dapagliflozin was not possible were also excluded.

### 2.2. Study Outcomes

The primary aim of the present study was to investigate factors (baseline characteristics at the time of administration of dapagliflozin) associated with patients in which loop diuretic dosage was reduced after initiating dapagliflozin. The dosage of diuretics (including whether or not the loop diuretic dosage was reduced) was evaluated before and 6 months after starting dapagliflozin treatment. Changes in the dosage of each diuretic were evaluated. Moreover, patients were divided into two groups in which the loop diuretic dosage was reduced or not, and each factor was compared. Multivariate analysis was performed using factors with significant differences between the two groups. In addition, a receiver operating characteristic (ROC) curve was created for the loop diuretic dosage at the time of initiating dapagliflozin administration to predict patients in whom the loop diuretic dosage could be reduced.

### 2.3. Data Collection

We evaluated the patient’s baseline clinical characteristics, medical history, comorbidities, physical findings, laboratory examinations, electrocardiographic findings, transthoracic echocardiographic findings (TTE), and medications and treatment information (including diuretics) using electronic medical records.

### 2.4. Patient Clinical Profiles

The severity of HF was assessed using the degree of symptoms (New York Heart Association (NYHA) classification) and brain natriuretic peptide (BNP). Previous HF, presence or absence of atrial fibrillation (AF), hypertension (HT), diabetes mellitus (DM), and chronic kidney disease (CKD) were investigated as medical history and comorbidities. HT, DM, and CKD were assessed based on medication history or according to respective guidelines. Previous HF was defined as a history of previous hospitalization for HF. In addition, underlying heart diseases such as ischemic heart disease and valvular disease were evaluated. As cardioprotective medications, we evaluated beta-blockers (BBs), renin–angiotensin–aldosterone system inhibitors (RAAS-Is), and mineralocorticoid receptor antagonists (MRAs). Angiotensin-converting enzyme inhibitors, angiotensin II type 1a receptor blockers or angiotensin receptor neprilysin inhibitor (ARNI) were considered RAAS-Is. MRAs were also evaluated as diuretics, along with loop diuretics, thiazides, and tolvaptan (TLV). The dosage of loop diuretics was also evaluated before and 6 months after starting dapagliflozin treatment. The dose of loop diuretic was calculated in terms of oral furosemide equivalent (oral furosemide 20 mg is equivalent to oral azosemide 30 mg, and oral furosemide 20 mg is equivalent to intravenous injection of furosemide 10 mg.).

### 2.5. Other Clinical Examinations

In laboratory examinations, liver function, renal function (including estimated glomerular filtration rate (eGFR)), hemoglobin, electrolytes, and brain natriuretic peptide (BNP) were evaluated.

The eGFR was calculated with the following formula: eGFR = 194 × Cr − 1.094 × age − 0.287 for men and 194 × Cr − 1.094 × age − 0.287 × 0.739 for women [15]. Cardiac size, wall thickness, and left ventricle systolic function (ejection fraction: EF) from TTE performed by two physicians blinded to the present study were analyzed. We calculated the EF using either the modified Simpson method (apical two- or four-chamber view) or the Teichholz method (parasternal long-axis view) [16], and we also assessed the proportion of patients with HF with preserved EF (HFpEF), defined as EF > 50% [17]. Also, 12-lead electrocardiogram was performed simultaneously with the TEE.

### 2.6. Statistical Analysis

Data are presented as means ± standard deviation or median. We used the Mann–Whitney U test to compare between two groups. Multivariate analysis from factors found to be significant upon comparison between two groups was investigated. *p* < 0.05 was considered statistically significant in all analyses. The ROC curves were analyzed to determine the cut-of value of the dosage of loop diuretics at the time of SGLT2 inhibitor initiation for predicting loop diuretic reduction. In addition, comparison of the dosage of diuretics was evaluated using Wilcoxon-signed-rank-test. We used EZR (Saitama Medical Center, Jichi Medical University), which is a graphical user interface for R (version 2.13.0, The R Foundation for Statistical Computing, Vienna, Austria) for the statistical analyses [18].

## 3. Results

### 3.1. Changes in the Dosage of Diuretics After Administration of Dapagliflozin

The median of loop diuretics dosage (oral furosemide equivalent) at the time of dapagliflozin administration was 20 mg/day (the mean dosage; 29.5 ± 26.5 mg/day), and after 6 months it decreased to 10 mg/day (the mean dosage; 14.5 ± 15.9 mg/day), (*p* < 0.001). In the reduced loop diuretics group (the R group), the median of loop diuretics dosage (oral furosemide equivalent) was significantly decreased (20 mg/day (the mean dosage; 34.1 ± 29.9 mg/day) to 10 mg/day (the mean dosage; 9.3 ± 12.7 mg/day), (*p* < 0.001); on the other hand, the median of loop diuretics in the not loop reduced group (the N group) have significantly difference (oral furosemide equivalent) (20 mg/day (the mean dosage; 21.5 ± 16.6 mg/day) to 20 mg/day (the mean dosage; 23.8 ± 17.1 mg/day), *p* = 0.028). In addition, of the 69 patients in the R group, 33 were able to discontinue loop diuretics. Forty patients (31.7%) were taking TLV, and the median dosage was reduced from 7.5 mg/day (the mean dosage; 8.16 ± 5.88 mg/day) to 3.75 mg/day (the mean dosage; 6.19 ± 6.10 mg/day) (*p* = 0.002). Thiazides were taken by 11 patients (8.7%), the majority of whom took trichlormethiazide, and the dosage did not change. MRA was administered to 85 patients (67.5%), and all took spironolactone. In five patients, treatment was discontinued due to hyperkalemia or other reasons, and the mean dosage of other patients did not change (25 mg/day (the mean dosage; 33.3 ± 14.3 mg/day) to 25 mg/day (the mean dosage; 34.8 ± 15.1 mg/day) (*p* = 0.949). Additionally, among patients where administration had not been performed due to hyperkalemia or other reasons, it was possible to start administration in five patients. Finally, for 126 patients, some kind of diuretic use was reduced, except for in 6 patients (4.8%). Furthermore, throughout the follow-up period, no patients were forced to discontinue SGLT2 inhibitors due to side effects such as hypotension.

### 3.2. Patient Backgrounds and Medications at Baseline

Patient characteristics at baseline are shown in Table 1. There were no differences in age, gender, and comorbidities; however, previous HF was more common in the R group, compared with the N group. Moreover, there were no differences in underlying heart disease between the two groups. There were also no differences in BNP or HYHA (Table 1; Table 2), and the severity of HF at the time of initiating dapagliflozin was similar between both groups.

With regard to the medications administered, BBs were significantly more prevalent in the N group (Table 3), which may reflect a higher previous HF in the N group. No significant differences were noted in pulse rate, despite differences in the frequency of BB use (Table 1). Systolic blood pressure (BP) also showed no difference. There were no differences in other cardioprotective medications, and no differences were observed between the two groups in the % patients on the three basic HF medications excluding SGLT2 inhibitors (the R Group: 42.0%, the N Group: 50.9%, *p* = 0.394) (Table 3). The rate of administration of loop diuretics in the R group was significantly higher, and the dosage of loop diuretics (oral furosemide equivalent) was also significantly higher compared with the N group (the R Group: 20 mg/day, the N Group: 10 mg/day, *p* < 0.001) (Table 3). However, no significant differences were observed for other diuretics. In addition, the proportion of patients who initiated dapagliflozin during hospitalization was significantly higher in the R group (50.5% vs. 14.0%, *p* < 0.001).

### 3.3. Clinical Examinations Between the Two Groups

Table 2 and Table 4 show laboratory and physiological examinations at the time of the initiation of dapagliflozin. There were no significant differences between the two groups in laboratory examination findings such as liver function and renal function. In addition, TTE showed no difference in cardiac chamber diameter between the two groups. EF was slightly decreased between the two groups; however, there was no difference (42.8% in the R Group, 49.2% in the N Group, *p* = 0.568), and the proportion of HFpEF was also not different (39.1% in the R Group, 47.4% in the N Group, *p* = 0.138). In electrocardiography at the time of 12-lead electrocardiogram, heart rate (HR) was significantly higher in the R group (83 bpm in the R group, 70 bpm in the N group, *p* = 0.001); however, 62 AF patients were included (49.2%).

### 3.4. Multivariate Analysis for Predicting Dose Reduction in Loop Diuretics

**Figure 1:**
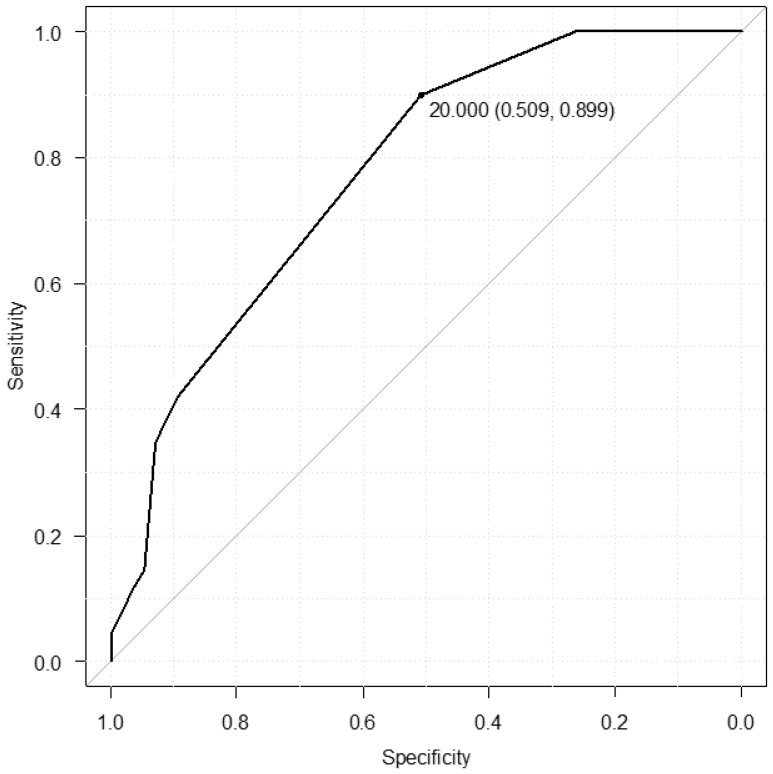
Receiver operating characteristic curve of the dosage of loop diuretics at baseline for predicting dose reduction in loop diuretics. The mean area under the ROC curve and 95% CI for the dosage of loop diuretics at baseline was 0.774 (95%CI 0.697–0.851). The cut-off value for loop diuretics at the time of initiating dapagliflozin based on the ROC curve was 20 mg/day, with a sensitivity of 0.509 and specificity of 0.899.

Multivariate analysis was performed on the four factors that showed significant differences in the above between two group comparisons: medical history of HF, in-hospital start of dapagliflozin, % patients on β-blockers, and the dosage of loop diuretics (HR by electrocardiogram showed a significant difference; however, since the pulse rate at the start of dapagliflozin showed no difference, it was not included in the multivariate analysis due to inconsistent results). Multivariate analysis showed that the three factors of in-hospital start of dapagliflozin, % patients on β-blockers, and the dosage of loop diuretics independently predicted the reduction in loop diuretic dosage (Table 5). The cut-off value from the ROC curve was 20 mg/day of loop diuretics (oral furosemide equivalent) at the time of initiation of dapagliflozin (area under the curve (AUC): 0.774, 95% confidence interval (CI): 0.697–0.851, sensitivity: 0.509, specificity: 0.899, Figure 1). The number of patients taking 20 mg/day or more of loop diuretics (oral furosemide equivalent) at the time of initiation of dapagliflozin was 62 patients (89.9%) in the R group and 28 patients (49.1%) in the N group. Seven patients in the R group who were taking 10 mg/day loop diuretic (oral furosemide equivalent) at the time of initiation of dapagliflozin were eventually able to discontinue the loop diuretic (oral furosemide equivalent).

The dosage of diuretics is often increased during hospitalization compared to outpatient care. Therefore, we performed a similar additional analysis on 83 patients, excluding those who started diuretics hospitalization. In the comparison between the two groups, in addition to the dosage of loop diuretics, significant differences were observed in af, sodium, and % patients on tolvaptan. Also, in the multivariate analysis excluding patients who initiated diuretics during hospitalization, the dosage of loop diuretics independently predicted a reduction in loop diuretics (Table 6). Also, in patients excluding those who initiated diuretics during hospitalization, the cut-off value for loop diuretics (oral furosemide equivalent) was also 20 mg/day (AUC: 0.747, 95%CI: 0.648–0.846, sensitivity: 0.562, specificity: 0.818).

## 4. Discussion

### 4.1. Main Findings

The introduction of dapagliflozin reduced the dosage of diuretics in the present study, a result similar to many previous studies [11,19]. One previous study, which evaluated diuretic dosage changes after starting SGLT2 inhibitors, revealed that 74% of patients did not change their diuretics, and 24% were able to reduce or discontinue diuretics [20]. However, no mention was made of the change in mean dose in this previous study. In the present study, when comparing the reduced loop diuretics and not reduced group, the dosage of loop diuretics was significantly reduced in the reduced loop diuretics group; however, it was significantly increased in the not reduced group. Multivariate analysis revealed that the dosage of loop diuretics independently predicted the reduction in loop diuretic dosage, and this result was similar even when excluding patients in which dapagliflozin was initiated during hospitalization. The cut-off value from the ROC curve was 20 mg/day of loop diuretics (oral furosemide equivalent) at the time of initiation of dapagliflozin. On the other hand, four patients in the R group who were taking 10 mg/day loop diuretics (oral furosemide equivalent) at the time of initiation of dapagliflozin were eventually able to discontinue the loop diuretic.

### 4.2. Heart Failure and Diuretics

HF presents with clinical symptoms such as dyspnea, edema, and decreased exercise tolerance, which are included in the Framingham diagnostic criteria [12]. Therefore, treatment is required to alleviate the symptoms associated with congestion, and diuretics and vasodilators are used, especially in the acute phase, according to the Clinical Scenario (CS) classification [16]. On the other hand, chronic diuretic use increases the long-term mortality rate and re-hospitalization rate in patients with HF [21]. The general consensus is that congestion should not be tolerated at discharge [22], and diuretics are often increased during hospitalization compared to outpatient care. Even during hospitalization in the acute phase of HF, the use of high-dose diuretics has been reported to be associated with poor prognosis, including increased mortality [23]. Therefore, it is recommended that diuretics be used at low doses during both hospitalization and outpatient care. In patients with stable chronic HF, short-term diuretic withdrawal is said to not cause worsening of symptoms [24]; however, in order to continuously reduce or withdraw diuretics over the long term, it is necessary to consider the use of other medications in combination. One of these medications is SGLT2 inhibitors. SGLT2 inhibitors have a water diuretic effect due to osmotic diuresis associated with urinary glucose excretion; thus, it mainly reduces intracellular fluid compared to extracellular fluid [25]. Addition of an SGLT2 inhibitor to a loop diuretic increases the natriuresis of the loop diuretic by 36% [26]. SGLT2 inhibitors suppress Na reabsorption by antagonizing the expression of SGLT2 in the proximal tubule, which is elevated in HF. As a result, the intraluminal Na concentration in the downstream loop of Henle increases, and the loop diuretic inhibits the enhanced Na-K-2Cl co-transporter 2, resulting in a synergistic effect [9]. In the present study, the introduction of dapagliflozin also reduced the average dosage of loop diuretics after 6 months. It was also revealed that the effect of dapagliflozin differs depending on the dosage of loop diuretic used at the time of initiation of dapagliflozin. One factor behind this result may be that loop diuretics have a different mechanism of action from SGLT2 inhibitors, as they primarily reduce extracellular fluid. Similarly to the SGLT2 inhibitor, TLV has a water diuretic effect that primarily reduces intracellular fluid, and a comparison of the effects of three medications (TLV, loop diuretics, and SGLT2 inhibitors) on HF has also been reported [27]. Due to the small number of patients included in the present study, it was not possible to determine which combination therapy is effective. In addition, the diuretic effect of SGLT2 inhibitors depends on blood glucose levels [28]. In light of these results, future research is required to determine which diuretic medications are more effective when used in combination with SGLT2 inhibitors. 

### 4.3. Multifaceted Effects of SGLT2 Inhibitors and Diuretics in HF

SGLT2 inhibitors have been shown to have multifaceted effects on HF. Among these, diuretics, sympathetic nerve suppression, myocardial energy metabolism improvement, renal protection, and BP reduction play important roles [29]. The use of loop diuretics for HF can worsen renal function due to reduced renal blood flow [30]; however, SGLT2 inhibitors do not worsen renal function. In addition, renal protective effects have been shown in CKD [31]. In this manuscript, the SGLT2 inhibitor demonstrated cardiac and renal protection; however, approximately 10% of the study subjects had HF and approximately 40% were taking diuretics. The concomitant use of SGLT2 inhibitors in HF patients using loop diuretics has been shown to reduce urinary renal tubular biomarkers [32], which is one of the reasons for renal protection. Most of the subjects in the present study had CKD, and the eGFR was in the 40% range. The combination of SGLT2 inhibitors with loop diuretics in such patients may be effective. Furthermore, when MRA, which is one of the cardioprotective medications for chronic HF, is used in patients with renal impairment, attention should be paid to the increase in K. The combination of MRA with an SGLT2 inhibitor suppresses the increase in K compared to MRA alone [33]; thus, the combination of an SGLT2 inhibitor is also effective in this respect. The use of a loop diuretic also activates the RAS and sympathetic nerves, and sympathetic nerve activation in HF is associated with prognosis [34]. We have reported that the SGLT2 inhibitor suppresses the cardiac sympathetic nerve [35], and the combination of dapagliflozin in the present study may have improved cardiac sympathetic nerves; however, this could not be evaluated in the present study because it was a retrospective study.

### 4.4. Effect of SGLT2 Inhibitor on BP in Patients with HF

In addition to cardiac function, vascular function is involved in the pathology of HF [36]. In the Japanese guidelines, it is recommended that treatment should be selected focused on BP based on CS classification, during the acute phase of HF [16]. It has been reported that BP at the time of discharge from hospital for acute HF is related to prognosis [37], because lowering BP reduces afterload and improves prognosis. A review of previous HF studies in which standard HF treatment was introduced showed that the introduction of SGLT2 inhibitors reduces BP [38]. The introduction of SGLT2 inhibitors reduced BP even in hypertensive patients receiving multi-medication therapy for resistant hypertension [39]. On the other hand, although an excessive reduction in BP leads to poor prognosis of HF [40], the additional administration of SGLT2 inhibitors in HF patients reduced systolic BP by approximately 2 mmHg [41] and did not cause an excessive reduction in BP. In the patients included in the present study, it was not possible to follow up the course of BP in all patients; however, no patients required discontinuation of SGLT2 inhibitors due to hypotension.

## 5. Study Limitations

The present study was a single-center retrospective study and was limited by the small number of target HF subjects. In Japan, two SGLT2 inhibitors, dapagliflozin and empagliflozin, are recommended for the treatment of HF [15]. The choice of which of these two medications to choose was left to the discretion of the physician. Therefore, to ensure a sufficient number of patients for analysis, we performed the analysis regardless of whether the treatment was started as outpatient care or during hospitalization, and we confirmed that the results were similar even when patients who started treatment during hospitalization were excluded. However, there were differences in the evaluation items required for the multivariate analysis, such as a medical history of HF and AF, and we were unable to secure a sufficient number of patients for analysis using only sinus rhythm patients. In addition, because the present study was a retrospective study, the extracted data were limited. Therefore, we were unable to evaluate quality of life, living environment (including salt intake), medication compliance, and follow up data. In addition, the dosage of loop diuretics was left to the discretion of each attending physician. These potential biases may undermine the strength of the conclusions. Further prospective clinical trials in larger consecutive subjects are needed to confirm our results.

## 6. Conclusions

In many patients, the additional administration of an SGLT2 inhibitor to HF patients allowed for a reduction in the dosage of diuretics. The cut-off value from the ROC curve was 20 mg/day of loop diuretics (oral furosemide equivalent) at the time of initiation of dapagliflozin; however, there were also patients where treatment was able to be discontinued even at 10 mg/day. After starting SGLT2 inhibitors in HF patients, it is important to consider reducing the dosage of loop diuretics.

## Figures and Tables

**Table 1:** Patient characteristics between groups.

	Loop Diuretics Reduced Group R Group (n = 69)	Loop Diuretics Not Reduced Group N Group (n = 57)	*p* Value
Age (years)	73	74	0.799
Male (n (%))	54 (78.3)	36 (63.2)	0.145
NYHA class (I/II/III/IV)	22/27/18/2	18/29/6/4	0.560
Systolic BP at administration of SGLT2-inhibitor (mmHg)	116	117	0.144
Pulse rate at administration of SGLT2-inhibitor (bpm)	79	71	0.626
In hospital start of dapagliflozin (n (%))	35 (50.7)	8 (14.0)	<0.001
Medical history of heart failure (n (%))	33 (47.8)	43 (75.4)	0.007
Medical history of atrial fibrillation (n (%))	38 (55.1)	24 (42.1)	0.211
Medical history of hypertension (n (%))	37 (53.6)	32 (56.1)	0.808
Medical history of diabetes (n (%))	36 (52.2)	23 (40.4)	0.254
Medical history of chronic kidney disease (n (%))	36 (52.2)	32 (56.1)	0.382
Ischemic heart disease (n (%))	17 (24.6)	21 (36.8)	0.239
Valvular heart disease (n (%))	28 (40.6)	27 (47.4)	0.512

NYHA: New York Heart Association, BP: blood pressure, SGLT2 inhibitor: sodium glucose cotransporter 2 inhibitor. Continuous data are expressed as the median. *p*-values were determined using the Mann–Whitney U test.

**Table 2:** Laboratory examinations between the two groups.

	Loop Diuretics Reduced Group R Group (n = 69)	Loop Diuretics Not Reduced Group N Group (n = 57)	*p* Value
Sodium (mEq/L)	140	140	0.121
Potassium (mEq/L)	4.2	4.1	0.980
Total bilirubin (mg/dL)	0.8	0.7	0.314
Aspartate aminotransferase (IU/L)	27	25	0.255
Alanine aminotransferase (IU/L)	19	16	0.115
Blood urea nitrogen (mg/dL)	20	21	0.560
Creatinine (mg/dL)	1.12	1.05	0.165
eGFR (ml/min/1.73 m^2^)	44.6	46.2	0.380
Hemoglobin (g/dL)	13.4	12.4	0.485
Brain natriuretic peptide (pg/mL)	533.8	451.3	0.207

eGFR: estimated glomerular filtration rate. Continuous data are expressed as the median. *p*-values were determined using the Mann–Whitney U test.

**Table 3:** Medications between the two groups.

	Loop Diuretics Reduced Group R Group (n = 69)	Loop Diuretics Not Reduced Group N Group (n = 57)	*p* Value
% patients on β-blockers (n (%))	50 (72.5)	54 (94.7)	0.031
% patients on angiotensin-converting enzyme inhibitors (n (%))	18 (26.1)	17 (29.8)	0.719
% patients on angiotensin II type 1a receptor blockers (n (%))	23 (33.3)	24 (42.1)	0.398
% patients on angiotensin receptor neprilysin inhibitor (n (%))	15 (21.7)	8 (14.0)	0.458
% patients on RAAS-Is (n (%))	55 (79.8)	49 (86.0)	0.547
% patients on MRAs (n (%))	52 (75.4)	33 (57.9)	0.092
% patients on β-blockers, RAAS-Is, and MRAs (n (%))	29 (42.0)	26 (50.9)	0.394
% patients on loop diuretics (n (%))	69 (100)	42 (73.7)	0.011
The median dosage of oral furosemide equivalent (mg/day)	20	10	<0.001
% patients on thiazide (n (%))	5 (7.3)	6 (10.5)	0.752
% patients on tolvaptan (n (%))	22 (31.9)	16 (28.1)	0.713

RAAS-I: renin–angiotensin–aldosterone system inhibitor, MRA: mineral corticoid receptor antagonist. Continuous data are expressed as the median. *p*-values were determined using the Mann–Whitney U test.

**Table 4:** Clinical physiological examinations between the two groups.

	Loop Diuretics Reduced Group R Group (n = 69)	Loop Diuretics Not Reduced Group N Group (n = 57)	*p* Value
Left atrial diameter (mm)	43.2	44.3	0.746
Left ventricular end-diastolic diameter (mm)	56.1	56.7	0.983
Left ventricular end-systolic diameter (mm)	43.0	41.1	0.759
Left ventricular ejection fraction (%)	42.8	49.2	0.568
The percentage of HFpEF (n (%))	27 (39.1)	27 (47.4)	0.813
Heart rate of electrocardiogram (bpm)	83	70	0.001
Atrial fibrillation (n (%))	38 (55.1)	24 (42.1)	0.211
QRS duration (ms)	98	102	0.275
QTc duration (ms)	437	439	0.153

HFpEF: heart failure with preserved ejection fraction. Continuous data are expressed as the median. *p*-values were determined using the Mann–Whitney U test.

**Table 5:** Multivariate analysis for predicting dose reduction in loop diuretics in all subjects.

	HR	95%CI	*p* Value
Medical history of heart failure	0.62	0.25–1.56	0.313
In hospital start of dapagliflozin	2.95	1.12–7.80	0.029
% patients on β-blockers	0.21	0.05–0.84	0.027
The dosage of oral furosemide equivalent	1.05	1.02–1.08	0.003

The multivariate analysis was performed applying Cox proportional hazards models. HR: hazard ratio, CI: confidence interval.

**Table 6:** Multivariate analysis for predicting dose reduction in loop diuretics in subjects excluding patients who initiated diuretics during hospitalization.

	HR	95%CI	*p* Value
Atrial fibrillation	2.93	1.07–8.06	0.037
Sodium	0.90	0.80–1.02	0.107
The dosage of oral furosemide equivalent	1.05	1.01–1.08	0.014
% patients on tolvaptan	0.42	0.10–1.74	0.233

The multivariate analysis was performed applying Cox proportional hazard models. HR: hazard ratio, CI: confidence interval.

## Data Availability

The datasets used and/or analyzed during the current study are available from the corresponding author on reasonable request.
